# Amphetamine Fails to Alter Cued Recollection of Emotional Images: Study of Encoding, Retrieval, and State-Dependency

**DOI:** 10.1371/journal.pone.0090423

**Published:** 2014-02-27

**Authors:** Jessica Weafer, David A. Gallo, Harriet de Wit

**Affiliations:** 1 Department of Psychiatry and Behavioral Neuroscience, University of Chicago, Chicago, Illinois, United States of America; 2 Department of Psychology, University of Chicago, Chicago, Illinois, United States of America; Tokai University, Japan

## Abstract

Stimulant drugs facilitate both encoding and retrieval of salient information in laboratory animals, but less is known about their effects on memory for emotionally salient visual images in humans. The current study investigated dextroamphetamine (AMP) effects on memory for emotional pictures in healthy humans, by administering the drug only at encoding, only at retrieval, or at both encoding and retrieval. During the encoding session, all participants viewed standardized positive, neutral, and negative pictures from the International Affective Picture System (IAPS). 48 hours later they attended a retrieval session testing their cued recollection of these stimuli. Participants were randomly assigned to one of four conditions (N = 20 each): condition AP (20 mg AMP at encoding and placebo (PL) at retrieval); condition PA (PL at encoding and AMP at retrieval); condition AA (AMP at encoding and retrieval); or condition PP (PL at encoding and retrieval). Amphetamine produced its expected effects on physiological and subjective measures, and negative pictures were recollected more frequently than neutral pictures. However, contrary to hypotheses, AMP did not affect recollection for positive, negative, or neutral stimuli, whether it was administered at encoding, retrieval, or at both encoding and retrieval. Moreover, recollection accuracy was not state-dependent. Considered in light of other recent drug studies in humans, this study highlights the sensitivity of drug effects to memory testing conditions and suggests future strategies for translating preclinical findings to human behavioral laboratories.

## Introduction

Drug addiction is thought to be a disorder of learning and memory [1], [2]. This hypothesis originated in part from evidence that drugs of abuse act directly on emotional memory systems that guide reward-related learning [3], [4]. In a natural state, such learning and memory are essential to direct behavioral pursuit of rewards necessary for survival (e.g., food, sex). However, drugs of abuse can activate these systems, heightening the significance of drug-rewards, resulting in maladaptive learning, and drug-seeking and drug-taking at the expense of other rewards. In this way, strong drug-related memories come to exert a substantial influence over the drug user's behavior. These influences are especially evident during relapse, when drug memories can elicit drug use even after long periods of abstinence [5], [6].

The effects of stimulant drugs on learning and memory for incentive stimuli have been studied in animal models, but less is known about their effects on memory for emotionally salient material in humans. Stimulants such as nicotine and amphetamine facilitate both encoding and retrieval of salient information in laboratory animals. That is, they enhance learning about environmental stimuli present at the time of administration (i.e., encoding), and they increase responding to conditioned reinforcers, which may indicate enhanced retrieval (for a review, see [4]). In contrast, we know of only two published studies that have examined stimulant effects on emotional memory in humans. In a previous study we assessed the effects of dextroamphetamine (AMP) and placebo administered during encoding of emotionally salient stimuli [7]. Retrieval was tested two days later, without the drug. AMP administered at encoding enhanced recognition memory accuracy relative to placebo, especially for emotional stimuli (both positive and negative), providing the first evidence that acute stimulant administration enhances encoding of emotionally salient material. In a second study [8] we assessed the effect of AMP administered during retrieval of emotional stimuli. Here, AMP did not affect recall or recognition accuracy, but it did increase memory intrusions including high-confidence errors. These effects were observed across all stimuli, and thus, contrary to AMP effects at encoding, were not larger for emotional stimuli.

In addition to directly affecting encoding or retrieval, the effects of drugs on memory may also depend on the concordance of drug states at the times of encoding and retrieval. In the preclinical literature a phenomenon of ‘drug state-dependency’ has been described in which information learned in one drug state is more readily recalled in the same drug state [9]. In humans, evidence for state-dependent memory has been mixed. State-dependency effects on explicit recall tasks have been reported for both memory-impairing drugs including marijuana and alcohol [10], [11], [12] and memory-facilitating stimulant drugs including methylphenidate and amphetamine [13], [14], [15]. Other studies have failed to observe state-dependent effects for these drugs [16], [17], [18], [19]. Importantly, to our knowledge no studies have examined the degree to which emotional memory is drug state-dependent. However, a related construct, known as mood-congruent memory, has been observed [20], [21]. That is, positive or negative information is remembered more quickly and accurately when individuals are in a mood state that is concordant with the information to be remembered [22], [23], although see the study by Eich [24]. These reports raise the possibility that emotional memory retrieval with stimulant drugs might be better when subjects are in a concordant drug state (i.e., both drug or both placebo) at encoding *and* retrieval. We examined this possibility here.

The current study had two aims: to test the effects of AMP on emotional memory in humans, and to determine whether emotional memory is state dependent. Based on our earlier work showing that AMP enhanced emotional memory, we chose complex images of emotional scenes as the to-be-remembered stimuli. Certain aspects of the procedure differed from our previous studies testing AMP effects on memory, mainly to test the state-dependency hypothesis. Specifically, we used a cued-recollection task instead of a recognition memory task, to specifically target drug effects on the explicit recollection of previously studied pictures. This type of memory task is advantageous to assess state-dependency of memory for several reasons. First, research indicates that cued recall is more sensitive to state-dependency effects than recognition [11], implicating a strong role for recollection in these effects. Whereas recognition memory involves both recollection and familiarity, and thus involves multiple subregions of the medial temporal lobe, cued recall more precisely targets hippocampal-mediated recollection [25]. Second, ceiling effects are less likely to be observed during cued recall compared to recognition, allowing for greater likelihood of observing drug-induced or state-dependent enhancement of performance. Finally, recollection is arguably more central to the concept of episodic memory than recognition [26], [27]. Given associations between episodic memory and drug abuse in humans [28], it is important to study drug effects on this type of memory.

An additional methodological difference between this and our previous studies was our decision to use a between-subjects design, in which we administered the drug only at encoding, only at retrieval, or at both encoding and retrieval. This design avoids any potentially confounding effects of task order, thus allowing for the clearest test of state-dependency. Subjects were randomly assigned to four conditions: in two of these, the drug state at encoding and retrieval were concordant (both drug or both placebo (PL)), and in the other two the drug states were discordant (drug at encoding only or drug at retrieval only). This allowed us to investigate drug effects at encoding and retrieval separately, and to examine drug-state dependency of memory. Based on our prior work described above, we hypothesized that AMP would be more likely to increase memory at encoding than at retrieval, and these drug effects would be greater for emotionally salient (i.e., positive and negative) than neutral material. Critically, we also predicted that memory would be greater when encoding and retrieval occurred in the same state (i.e., AMP-AMP or PL-PL) compared to different states (i.e., AMP-PL or PL-AMP).

## Methods

### Ethics statement

This study was approved by the Institutional Review Board of the University of Chicago and was carried out in accordance with the Declaration of Helsinki. All participants provided written informed consent for participation.

### Participants

Healthy volunteers (N = 80) were recruited from the community through online and printed advertisements. Inclusion criteria included age 18–35, BMI between 19 and 26, at least a high school education, fluency in English, no current or past year DSM-IV diagnosis (including substance abuse), no lifetime history of substance dependence, no serious medical conditions, and no night shift work. Females who were not on hormonal contraception were tested only in the follicular phase of their menstrual cycle [29].

### Design

This study utilized a two-session, double-blind, between-subjects design in which subjects attended an encoding session, followed by a retrieval session exactly 48 hours later. Equal numbers of men and women were randomly assigned to one of four conditions (N = 20 each): condition AP (20 mg AMP at encoding and PL at retrieval); condition PA (PL at encoding and AMP at retrieval); condition AA (AMP at encoding and retrieval); or condition PP (PL at encoding and retrieval; see Table 1). Participants viewed labels and pictures during the encoding session, and their memory for these stimuli was tested during the retrieval session. Physiological and subjective measures were recorded over 3.5 hours following drug administration.

**Table 1:** Encoding and retrieval drug by condition.

Condition (N = 20 each)	Encoding Session Drug	Retrieval Session Drug
AP	AMP (20mg)	PL
PA	PL	AMP (20mg)
AA	AMP (20mg)	AMP (20mg)
PP	PL	PL

*Note*. AMP  =  amphetamine; PL  =  placebo.

### Procedure

Participants first attended an orientation session in which they provided informed consent and were familiarized with laboratory procedures and study protocol. Participants were told that the study was investigating the effects of drugs on memory for emotional material, and to minimize drug expectancies they were told they could receive one of the following: stimulant, sedative, marijuana-like drug, or placebo. Participants practiced the study and test phases of the recollection task, as well as the subjective questionnaire measures. They were instructed to consume their normal amounts of caffeine and nicotine, but to abstain from drugs, including alcohol, for 24 hours prior to each session, and to not consume any food after 7am.

The experimental sessions took place from 9am to 1pm, and were separated by exactly 48 hours. Participants were tested individually. Upon arrival, compliance with drug abstinence was verified by both self-report and breath and urine screens (testing for amphetamine, cocaine, methamphetamine, opiates, and tetrahydrocannabinol). Baseline (pre-drug) physiological and subjective measures were obtained. At 9:30am, participants ingested opaque capsules that contained either drug or placebo. Subjective and physiological measures were assessed at 30, 60, 90, 150, and 180 min after capsule administration. Participants performed the recollection task (study phase on day 1 and test phase on day 2), beginning at 90 min after capsule administration. Participants left the laboratory at 1pm, after confirmation that physiological measures had returned to baseline. Once both experimental sessions were complete, participants were debriefed and compensated for their time.

### Measures

#### Cued recollection task

Memory was assessed using a cued recollection procedure (cf. [30]). The stimuli were 144 pictures drawn from the International Affective Picture System (IAPS; [31]), which consists of standardized positive, neutral, and negative pictures. Two or three word descriptive labels were created for each picture (e.g., ‘angry man face’, ‘sailboat on ocean’). From the total 144 pictures, two picture sets (A and B) were created (72 pictures each; 24 negative, 24 neutral, 24 positive, based on normative ratings with emotional items matched for arousal), and an equal number of subjects in each condition were assigned to set A or set B. During the encoding phase, all participants viewed each of the 144 labels (48 negative, 48 neutral, 48 positive) in random order. Then, for half of the labels (either Set A or B), the picture described by the label was presented on the screen. During the retrieval sessions, participants were again presented with all 144 labels, one at a time, and asked to say whether or not they remembered seeing a picture with that label.

#### Encoding phase

During the encoding phase, participants viewed the labels and pictures. Prior to each label, a fixation point was presented on the screen. Participants pressed the space bar and a label was presented for 1500 ms. After each label was presented, participants rated how likely they were to remember a picture associated with that label, on a scale from 1 ‘not at all likely’ to 5 ‘very likely’. Following half of the labels (either Set A or B), the picture associated with the label was presented on the screen for 2000 ms. Following presentation of each picture, participants rated the arousal and valence of the picture on three 5-point scales: affective valence (positive: 1 to 5; negative 1 to 5) and arousal (1 to 5).

#### Retrieval phase

Exactly 48 hours later, participants returned to the laboratory for the retrieval session. Participants performed the cued recollection test, in which they viewed the 144 labels from the encoding phase (72 had been associated with a picture, and 72 had not) in random order. For each label they were asked whether they remembered seeing a picture that was associated with the label or not (yes/no). They then rated how confident they were in their decision on a 5-point scale, ranging from ‘not at all’ to ‘extremely’. Because participants viewed all of the test labels during encoding, all of the labels should have been familiar to the participants. As such, familiarity with the test label alone was insufficient to perform above chance accuracy on this task. Instead, the ability to discriminate between targets and lures relied on accurate recollection of the studied pictures associated with the targets (and not lures).

### Subjective response measures

#### Drug Effects Questionnaire (DEQ)

The DEQ consists of three items on a visual analogue scale (0 to 100 mm) that measure subjective drug response. Participants rate the extent to which they ‘feel drug’, ‘like drug’, and ‘feel high’.

#### Profile of Mood States (POMS; [32])

The modified POMS consists of 72 adjectives commonly used to describe momentary mood states and has been factor analyzed into eight scales (Friendliness, Vigor, Anxiety, Fatigue, Elation, Depression, Anger, and Confusion). Participants indicate how they feel at the moment in relation to each adjective on a 5-point scale from ‘not at all’ (0) to ‘extremely’ (4). We focused our analyses on the Elation and Vigor scales, as these represent the typical positive, rewarding effects of amphetamine (e.g., [33], [34], [35]).

### Physiological effects measures

Blood pressure and heart rate were measured using a portable digital blood pressure monitor (AND Medical/Life Source, San Jose, CA).

### Data analysis

Three dependent variables were calculated from performance of the cued recollection task: hit rate (the proportion of labels correctly identified as studied with a picture); false alarm rate (the proportion of labels incorrectly identified as studied with a picture); and recollection accuracy (hit rate minus false alarm rate). For each dependent measure, analyses were conducted first by collapsing across picture valence, and then individually for positive, negative, and neutral stimuli. Conditions AP, PA, and AA were individually compared to condition PP in between-groups t tests to examine AMP effects at 1) encoding, 2) retrieval, and 3) encoding and retrieval. Additionally, conditions AP and PA were compared to conditions AA and PP to test if memory was state-dependent.

## Results

### Sample characteristics

Demographic information and current substance use for each of the four conditions is presented in Table 2. The groups did not differ on any measure, although the PP group included one heavy cannabis user. Exclusion of this participant did not change the outcome for any analyses, so results are reported with all participants included.

**Table 2:** Mean (SD) age, education, and drug use by condition (N = 20 each).

	AP	PA	AA	PP
Age (years)	22.9 (3.8)	24.4 (4.1)	24.0 (3.2)	24.2 (5.3)
Education (years)	14.9 (1.4)	14.9 (1.8)	15.5 (1.7)	14.2 (1.3)
Caffeine (cups/day)	0.9 (0.5)	1.6 (1.4)	1.3 (1.0)	1.6 (1.7)
Nicotine (cigarettes/week)	1.6 (5.6)	4.2 (12.8)	1.6 (4.2)	2.0 (7.8)
Alcohol (drinks/week)	6.0 (5.8)	8.0 (4.5)	7.4 (7.6)	7.7 (5.7)
Cannabis (times/month)	0.1 (0.2)	2.7 (5.9)	3.0 (7.0)	6.8 (23.5)

*Note.* AP = AMP at encoding, PL at retrieval; PA = PL at encoding, AMP at retrieval.

AA = AMP at encoding and retrieval; PP = PL at encoding and retrieval.

### Subjective and physiological effects

**Figure 1:**
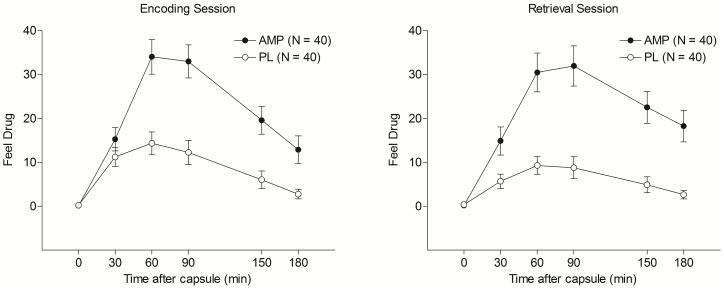
Mean ratings of Feel Drug following AMP and PL at baseline and 30, 60, 90, 150, and 180(left panel) and during the Retrieval session (right panel). Capped vertical lines indicate standard errors of the mean.

To ensure active pharmacological effects of AMP, we first examined subjective and physiological measures. Area under the curve (AUC) values were calculated for each measure during both the encoding and retrieval sessions, and these are presented in Table 3. Between groups t tests showed that AMP significantly increased subjective and physiological responses relative to PL on both sessions. Representative plots of the AUC values for ratings of Feel Drug following AMP and PL on the Encoding and Retrieval sessions are presented in Figure 1.

**Table 3:** Mean (SEM) area under the curve values for subjective and physiological measures.

	Encoding Session		Retrieval Session	
	AMP (N = 40)	PL (N = 40)	AMP (N = 40)	PL (N = 40)
DEQ				
Feel drug	4.2 (0.4)***	1.7 (0.3)	4.0 (0.5)***	1.2 (0.3)
Like drug	6.9 (0.6)***	3.6 (0.6)	6.6 (0.8)***	2.4 (0.5)
Feel high	2.7 (0.4)**	1.4 (0.3)	2.9 (0.5)***	0.8 (0.2)
POMS				
Elation	9.2 (10.6)**	−26.9 (6.2)	23.9 (7.9)**	−11.0 (6.0)
Vigor	13.8 (15.7)**	−39.1 (7.9)	47.3 (12.9)***	−28.1 (7.7)
Heart rate	43.8 (21.5)***	−56.6 (17.0)	12.6 (22.3)**	−82.5 (20.1)
Systolic blood pressure	243.2 (21.9)***	6.1 (16.2)	220.7 (17.8)***	−6.4 (13.1)
Diastolic blood pressure	155.5 (17.4)***	−8.7 (14.0)	139.4 (17.5)***	−11.8 (16.0)

*Note.* DEQ = Drug Effects Questionnaire. POMS = Profile of Mood States. ***p<0.001, **p<0.01, AMP compared to PL.

### Cued recollection task

**Figure 2:**
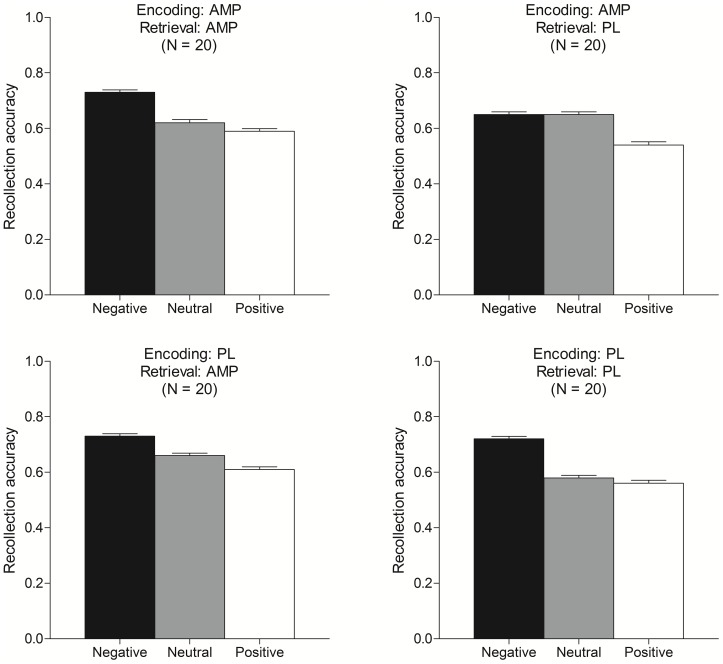
Mean recollection accuracy scores presented by condition for each picture valence (Neg  =  negative, Neu  =  neutral, and Pos  =  positive). Capped vertical lines indicate standard errors of the mean.

Mean recollection accuracy scores are presented by valence for each condition in Figure 2. Mean hit rate and false alarm rate for the individual picture valences and for all stimuli combined are presented by condition in Table 4 and by drug state in Table 5. Results were analyzed first using all responses, and then additionally using only high confidence responses in order to more clearly index picture recollection as opposed to a more vague feeling of familiarity elicited by the retrieval cues (cf. [36]). Similar results were obtained using both methods, and so the results presented here are derived from all responses.

**Table 4:** Mean (SEM) hit rate and false alarm rate by condition (N = 20 each).

	Hit Rate			
Condition	Negative	Neutral	Positive	All
AP	.73 (.03)	.70 (.04)	.70 (.04)	.71 (.03)
PA	.83 (.03)	.76 (.04)	.75 (.03)	.78 (.03)
AA	.82 (.03)	.71 (.05)	.73 (.04)	.75 (.04)
PP	.77 (.04)	.67 (.04)	.69 (.04)	.71 (.04)
All	.79 (.02)	.71 (.02)	.72 (.02)	.74 (.02)

	False Alarm Rate			
Condition	Negative	Neutral	Positive	All
AP	.08 (.02)	.05 (.01)	.16 (.02)	.10 (.02)
PA	.10 (.02)	.09 (.02)	.14 (.03)	.11 (.01)
AA	.10 (.02)	.09 (.02)	.14 (.02)	.11 (.01)
PP	.05 (.02)	.09 (.03)	.13 (.03)	.09 (.02)
All	.08 (.01)	.08 (.01)	.14 (.01)	.10 (.01)

*Note.* AP = AMP at encoding, PL at retrieval; PA = PL at encoding.

AMP at retrieval, AA = AMP at encoding and retrieval; PP = PL at encoding and retrieval.

**Table 5:** Mean (SEM) recollection accuracy, hit rate, and false alarm rate by drug state.

	Recollection Accuracy			
State	Negative	Neutral	Positive	All
Same	.72 (.03)	.60 (.03)	.58 (.03)	.63 (.03)
Different	.69 (.03)	.66 (.03)	.58 (.03)	.64 (.03)

	Hit Rate			
State	Negative	Neutral	Positive	All
Same	.80 (.03)	.69 (.03)	.71 (.02)	.73 (.02)
Different	.78 (.02)	.73 (.03)	.73 (.02)	.74 (.02)

	False Alarm Rate			
State	Negative	Neutral	Positive	All
Same	.08 (.01)	.09 (.02)	.13 (.02)	.10 (.01)
Different	.09 (.01)	.07 (.01)	.15 (.02)	.10 (.01)

*Note.* Same = same drug state (AA or PP) at encoding and retrieval.

Different = different drug state (AP or PA) at encoding and retrieval.

#### Emotional memory effects

Cued recollection task performance scores for positive and negative stimuli were individually compared to those for neutral stimuli to assess the degree to which the expected emotional effects on memory were observed. Paired t tests showed that both recollection accuracy and hit rate were greater for negative stimuli compared to neutral stimuli, ts(79)>4.6, ps<.001, and false alarm rate did not differ (p = .73). These effects replicate the typical emotional benefit on memory [37]. By contrast, recollection accuracy was worse for positive stimuli relative to neutral stimuli, t(79) = 3.0, p = .004, false alarm rate was greater, t(79) = 5.2, p<.001, and hit rate did not differ (p = .46). These emotional effects on false alarms also are consistent with prior work using this kind of cued recollection task [30], [38].

#### Encoding

AMP effects at encoding were analyzed by comparing conditions AP and PP. AMP did not affect recollection accuracy overall (p = .88) or when analyzed individually by valence (ps>.25). AMP did not affect hit rate or false alarm rate in the stimuli overall (ps>.73), or for any individual valence (ps>.24).

#### Retrieval

AMP effects at retrieval were analyzed by comparing conditions PA and PP. AMP at retrieval did not affect recollection accuracy overall (p = .33), or when analyzed individually by valence (ps>.13). AMP did not affect hit rate or false alarm rate in the stimuli overall (*p*s>.13), or for any individual valence (ps>.10).

#### Encoding and retrieval

AMP effects at encoding and retrieval were analyzed by comparing conditions AA and PP. AMP administered at both encoding and retrieval did not affect recollection accuracy overall (p = .62), or individually by valence (ps>.53). AMP did not affect hit rate or false alarm rate in the stimuli overall (ps>.37), or individually by valence (ps>.05).

#### State-dependency

Conditions AA and PP (same state) were compared to AP and PA (different state) to test if memory was state-dependent. Recollection accuracy did not differ overall (p = .84), or when analyzed individually by valence (ps>.19). Hit rate and false alarm rate did not differ in the stimuli overall (ps>.70), or individually by valence (ps>.34).

### Supplemental analyses

Additional analyses were conducted to test AMP effects on ratings of picture valence and arousal, and on memorability by comparing all participants who received AMP at encoding (conditions AP and AA; N = 40) to participants who received PL at encoding (conditions PA and PP; N = 40). Negative pictures were rated as more negative following AMP (mean valence rating = −2.3) compared to PL (mean valence rating = −1.8), t(78) = 3.0, p = .003. No other effects of AMP on picture ratings were significant (ps>.08). AMP effects on confidence ratings during the memory test were examined by comparing participants who received AMP at retrieval (conditions PA and AA; N = 40) to participants who received PL at retrieval (conditions AP and PP; N = 40). No significant drug effects were observed (ps>.56).

## Discussion

This study examined the effect of AMP and drug state-dependency on memory for emotional material in healthy humans. Contrary to our hypotheses, AMP did not affect memory for either emotional or neutral stimuli, when administered at encoding, at retrieval, or at both encoding and retrieval. Additionally, memory was not drug state-dependent, as no differences were observed between subjects who were in the same drug state at encoding and retrieval and those who were in different drug states. While it is possible that our task was insensitive to drug effects on memory, we did replicate the typical emotional enhancement of memory for negative pictures [37], and we also replicated prior findings of elevated false alarms to emotionally positive items in this kind of task [30], [38]. These findings demonstrate that our procedures were sensitive to detect memory differences. Moreover, AMP produced its expected effects of increased subjective ratings, heart rate, and blood pressure compared to PL, showing that the drug did have its usual pharmacological effects.

The current drug findings differ from those we have previously reported [7], [8], but there were several methodological differences. Perhaps the most important was that the specific memory tasks differed across studies. The memory tasks administered in our previous studies and the current study each used emotional stimuli drawn from the IAPS picture set. However, in most of our previous studies, memory for the stimuli has been tested with recognition tests (i.e., subjects view pictures and are asked if they recognize each picture). By contrast, the current drug study was the first to use a cued-recollection test procedure, in which participants were required to recollect pictures using verbal labels as retrieval cues. Based on prior work described in the Introduction, we reasoned that this cued recollection task would be more sensitive to drug-state dependency effects in memory than recognition memory tasks, which could be based on familiarity alone. However, in hindsight, it is possible that this cued recollection test introduced more strategic aspects to performance that made it less sensitive to drug effects on memory. For example, self-initiated attentional mechanisms may have been used to associatively bind the label and the pictures during encoding (cf. [39]) and strategic mental imagery processes may have been used to ‘trigger’ or self-cue memories during retrieval cf.[40], [41]. Future work will be needed to determine if these more strategic aspects of performance interact with drug effects. Also, the current study used a between-subjects drug design to provide a clearer test of state-dependent effects, but it is possible that a within-subjects design might have been more sensitive to drug effects on memory.

We predicted that memory would be drug-state dependent based on animal studies showing state-dependency and human studies showing both drug state-dependent and mood-congruent memory. Again, the current findings did not support this hypothesis, perhaps due to the specific memory task administered. It has been suggested that free recall is a more sensitive measure of drug state-dependent memory than cued recall, as the retrieval cues could potentially overpower the more subtle effects of drug cues [42]. Alternately, it is possible that the effects of AMP in the current study were not strong enough to produce either state-dependent or mood-congruent memory effects. This is an interesting issue that may be addressed in the future, using higher doses of drugs.

The current findings are inconsistent with some studies with laboratory animals that have reported stimulant drugs enhance both encoding and retrieval of salient information. One potentially important difference between the present study and the animal studies is that drugs in the animal studies are often administered chronically (e.g., [43], [44], [45]), raising the possibility that drug-induced neuroadaptations could contribute to drug effects on memory. To the extent that such neuroadaptations are necessary to observe drug effects on memory, the acute, one-time dose of AMP administered in the current study might have a smaller effect. Further, animal studies typically assess memory for conditioned stimuli that are explicitly paired with primary reinforcers, whereas our study, like many human studies, used pictures with emotional content as to-be-remembered stimuli. While these pictures possess some intrinsic affective content, based on normed valence ratings, they may be less salient than appetitive stimuli to rodents. It is also possible that participants' previous experiences could affect their memory for these types of stimuli. Finally, and perhaps most critically, our task specifically targeted episodic memory in humans, whereas the preclinical drug studies discussed potentially tapped into fundamentally different learning and memory systems in the brain [46].

In sum, the current study did not support our hypothesis that AMP would enhance emotional memory, or that its effects on memory are state-dependent. However, our findings do suggest alternate ways to test the effects of stimulant drugs on memory in humans. Specifically, recognition procedures used in our prior work are likely more sensitive measures of drug effects than the cued-recollection task used in the current study. Additionally, to more closely parallel animal studies, future studies could test drug effects on memory in regular stimulant users or abusers, as this would be more in line with the chronic drug administration procedures used in preclinical studies. Future studies in humans should also investigate drug effects on memory for previously neutral stimuli that have been conditioned in a controlled laboratory environment. This would minimize individual differences in prior associations with the emotional stimuli currently used in these paradigms, and again would more closely approximate methodologies used in animal studies. In sum, this study highlights the difficulty of translating preclinical findings to human behavioral laboratories, and suggests future strategies for such translational research.
